# Noise Trauma Induced Neural Plasticity Throughout the Auditory System of Mongolian Gerbils: Differences between Tinnitus Developing and Non-Developing Animals

**DOI:** 10.3389/fneur.2015.00022

**Published:** 2015-02-10

**Authors:** Konstantin Tziridis, Sönke Ahlf, Marcus Jeschke, Max F. K. Happel, Frank W. Ohl, Holger Schulze

**Affiliations:** ^1^Experimental Otolaryngology, Friedrich-Alexander University Erlangen-Nürnberg, Erlangen, Germany; ^2^Leibniz Institute for Neurobiology, Magdeburg, Germany; ^3^Institute of Biology, Otto-von-Guericke-University, Magdeburg, Germany; ^4^Center for Behavioral Brain Sciences, Magdeburg, Germany

**Keywords:** electrophysiology, auditory cortex, auditory brainstem, LFP, ABR

## Abstract

In this study, we describe differences between neural plasticity in auditory cortex (AC) of animals that developed subjective tinnitus (group T) after noise-induced hearing loss (NIHL) compared to those that did not [group non-tinnitus (NT)]. To this end, our analysis focuses on the input activity of cortical neurons based on the temporal and spectral analysis of local field potential (LFP) recordings and an in-depth analysis of auditory brainstem responses (ABR) in the same animals. In response to NIHL in NT animals we find a significant general reduction in overall cortical activity and spectral power as well as changes in all ABR wave amplitudes as a function of loudness. In contrast, T-animals show no significant change in overall cortical activity as assessed by root mean square analysis of LFP amplitudes, but a specific increase in LFP spectral power and in the amplitude of ABR wave V reflecting activity in the inferior colliculus (IC). Based on these results, we put forward a refined model of tinnitus prevention after NIHL that acts via a top-down global (i.e., frequency-unspecific) inhibition reducing overall neuronal activity in AC and IC, thereby counteracting NIHL-induced bottom-up frequency-specific neuroplasticity suggested in current models of tinnitus development.

## Introduction

Central, subjective tinnitus – the perception of a continuous phantom sound in the absence of any physical source – is a disorder with increasing prevalence (1) currently affecting 10–15% of the general adult population (2). Because tinnitus may lead to concentration problems, insomnia, anxiety, depression, and even the risk of suicide (3–5), a person’s ability to lead a normal life may be profoundly affected by the condition.

Despite these severe impairments and the large number of patients affected, there is still no established treatment available to safely and effectively alleviate tinnitus. The development of effective therapies for tinnitus is mainly hampered by the fact that the exact neuronal mechanisms leading to the phantom percept and its chronic manifestation are still largely unknown. Hence a significant amount of data from both human (6, 7) and animal studies (8–12) on the plastic changes occurring in the involved neuronal networks during tinnitus development is available, a number of different and in part controversial neurobiological models trying to explain the phenomenon do exist. These models may be divided into two main classes: Models of the first class propose that damage to the peripheral receptor epithelium of the cochlea leads to an imbalance of excitatory and inhibitory connections along the tonotopic gradient at multiple levels of the auditory pathway, finally resulting in a disinhibition of auditory neurons [e.g., Ref. (9, 13)]. Models of the second class propose a homeostatic response of the central auditory system trying to compensate for reduced cochlear input via an increased response gain [e.g., Ref. (14, 15)]. Furthermore, there may be different sub-types of tinnitus depending on the exact cause of the condition, e.g., the type and extent of cochlear damage. Finally, the exact relations between cochlear damage, hearing impairment, and tinnitus development are still elusive.

Although short or acute tinnitus percepts are experienced occasionally by virtually everyone, chronic tinnitus develops only in a sub-population of humans as well as in the different animal model systems. We currently do not know why some patients with hearing impairments develop chronic tinnitus while others with the same shift in hearing threshold do not, and vice versa why some but not all subjects without detectable noise-induced hearing loss (NIHL) still develop tinnitus, although first models that may solve this enigma have been published (14–17).

We have recently published the first report comparing neuronal plasticity following NIHL in animals that develop a tinnitus percept (group T) with plastic changes in animals that do not [group non-tinnitus (NT)], even though both animal groups did not differ in NIHL (17). As we could demonstrate, NT animals showed higher overall auditory cortical and brainstem activity before noise trauma compared to T-animals. We concluded that animals with low overall neuronal activity in the auditory system might have a predisposition for the development of tinnitus early after noise trauma while animals with higher neuronal activity did not develop such a percept within the time of observation. Furthermore, within a week after the acoustic trauma T-animals showed increased activity in auditory cortical neurons representing the tinnitus frequencies, whereas NT animals exhibited decreased activity at moderate sound intensities at that point in time. Spontaneous activity was generally increased in T but decreased in NT animals.

Based on these results we have proposed a model for tinnitus prevention that points to a global inhibitory mechanism in auditory cortex (AC) that may prevent tinnitus genesis in animals with high overall activity in the auditory system, whereas this mechanism does not seem to be available for tinnitus prevention in animals with low overall activity. We suggest that by activating this global inhibitory mechanism the NT animals may actively counteract the development of maladaptive increased response gains in AC seen in T-animals that possibly reflect the tinnitus development. According to this model, the high overall AC activity of NT animals before the trauma is a prerequisite for the activation of this hypothetical global inhibition. We suggest that a certain dynamic range for unspecific gain reduction across the whole AC is needed to successfully counteract the response gain increase that is associated with tinnitus development. If the overall neuronal activity in AC is already low before the trauma, as in the case of T-animals, this activity may not be reduced further and tinnitus develops because of un-resisted response gain increase in those regions of AC representing the tinnitus percept. What remains an open question in our model is the source of the hypothetical global inhibition, whether it is generated intra-cortically (within AC), sub-cortically or even in higher cortical or limbic structures.

In the present follow-up study we present a further, more detailed analysis compared to the Ahlf et al. study (17): by analyzing NIHL-induced plastic changes in local field potentials (LFP) reflecting mostly synaptic input activity in AC and comparing them to changes of auditory brainstem responses (ABR) in both T and NT animals we can further narrow down possible candidate structures for the source of global inhibition in the context of tinnitus prevention.

## Materials and Methods

### Ethics statement and animals

Mongolian gerbils (*Meriones unguiculatus*) were housed in standard animal racks (Bio A.S. Vent Light, Ehret Labor- und Pharmatechnik, Emmendingen, Germany) in groups of 2–3 animals per cage with free access to water and food at 20–24°C room temperature under 12/12 h dark/light cycle. The use and care of animals was approved by the state of Bavaria (Regierungspräsidium Mittelfranken, Ansbach, Germany). The authors declare that there is no conflict of interests regarding the publication of this paper.

A total of 35 eight to ten weeks old male gerbils purchased from Charles River Laboratories, Inc. (Sulzfeld, Germany) were used in this study. All methods used in this paper (except the LFP recordings) have been described previously (17) but still will be explained in detail here for better intelligibility.

### Time regime and overview of behavioral and ABR measurements

All animals were handled before the beginning of the experiments and accustomed to the setup environment to minimize stress. During the first week, animals were tested first, with three different paradigms of pre-pulse inhibition (PPI) of the acoustic startle reflex (ASR) to evaluate the audiograms for each animal and acquire baseline data for the tinnitus percept evaluation. Second, ABR measurements of the healthy animals were performed to obtain individual audiograms under anesthesia and the far field responses of the auditory pathway in the brainstem up to the inferior colliculus (IC). After these measurements surgery for implantation of the head holder and the recording chamber over the left AC was performed and electrophysiological single electrode recordings in primary AC field AI of the healthy anesthetized animal were carried out on two to three sessions within 1 week after the surgery. Acoustic trauma (2 kHz, 115 dB SPL, 75 min) under deep anesthesia was used to induce a mild NIHL (evaluated by ABR analysis directly after the trauma and PPI audiogram after 1 week) and a persistent tinnitus percept in the majority of the animals (26 of 35 animals, 74%; evaluated by PPI measurement one week after trauma). Within the first week after the trauma all electrophysiological recordings in AC were performed in two to three recording sessions. All experiments were carried out in an IAC (Industrial Acoustics Company GmbH, Niederkrüchten, Germany) acoustic chamber on a TMC (Technical Manufacturing Corporation, Peabody, MA, USA) low-vibration table.

The setups for behavioral and electrophysiological recordings have been described in detail earlier (17, 18). Briefly, for behavioral testing, animals were placed in a transparent acrylic tube (length: 10 cm; inner diameter 4.3 cm). This tube was placed 10 cm in front of a speaker (Canton Plus X Series 2) onto a Honeywell FSG15N1A piezo force sensor (sensitivity 0.24 mV/gram; null shift at ±25°C is ±1 mV; force range 0–1500 g), assembled within an IAC acoustic chamber on a TMC low-vibration table. The front end of the tube was closed with a stainless steel grate (wire mesh width 0.5 mm) allowing acoustic stimulation with no detectable distortion within the used stimulation range of 250–16000 Hz (signal to noise ratio at least 70 dB). Sound pressure level was controlled via a B&K Type 2610 measuring amplifier fed with a B&K Type 2669 preamplifier/B&K Type 4190 condensor microphone combination. Stimulus generation and data acquisition was controlled using custom-made Matlab 2008 programs (MathWorks, Natick, MA, USA; stimulation/recording sampling rate 20 kHz). For sound generation, the frequency response function of the speaker was calibrated to produce an output spectrum that was flat within ±1 dB measured within the acrylic tube.

Three different types of PPI modulated ASR paradigms (17) were performed to assess, first, hearing capacities [behavioral and reflex based audiogram (19)] and, second, the potential existence of a tinnitus percept (20) after the noise trauma. The measurements were performed before and usually 7 days after the acoustic trauma in a single 4 h block. For obtaining the behavioral hearing thresholds we used a PPI of ASR paradigm in all animals. We startled the animals with 90 dB SPL pure tones (6 ms length including 2 ms rise and fall ramps) ranging from 0.5 to 16 kHz in a pseudo-randomized order in octave steps. The same pure tones were used as pre-stimulus probes ranging from 0 to 50 dB SPL in 10 dB steps 100 ms before the startle stimulus. Each combination of pure tone frequency and pre-stimulus intensity was repeated 15 times. This procedure was performed before the acoustic trauma and one week after. The data obtained were checked by eye via a custom-made Matlab program, trials in which the animals moved within 100 ms before the startle stimulus were discarded; in the valid trials only peak-to-peak amplitudes of responses within the first 50 ms after startle stimulus onset were used for further analysis. The evaluation was performed independently by the principal investigator and a technical assistant, who was blind to the state of the animal. Evaluations of both experimenters led to identical results. We made sure that the animals were always (pre and post trauma = trauma status) responding to the 90 dB SPL startle stimulus (cf. 0 dB pre-stimulus response in Figure S1 in Supplementary Material). For validation of the PPI effect of the pre-stimuli we performed 1-factorial ANOVAs for the valid response amplitudes dependent on the pre-stimulus intensity for each frequency and trauma status separately for each individual animal. The mean responses before and after trauma of the six animals used for electrophysiological recordings in AC are given in Figure S1 in Supplementary Material. Responses in this threshold paradigm were fitted with a sigmoidal Boltzmann function for each frequency, trauma status, and animal separately. Hearing thresholds were defined as the sound level at the inflection point of the Boltzmann function for each frequency before and after trauma (18).

**Figure 1:**
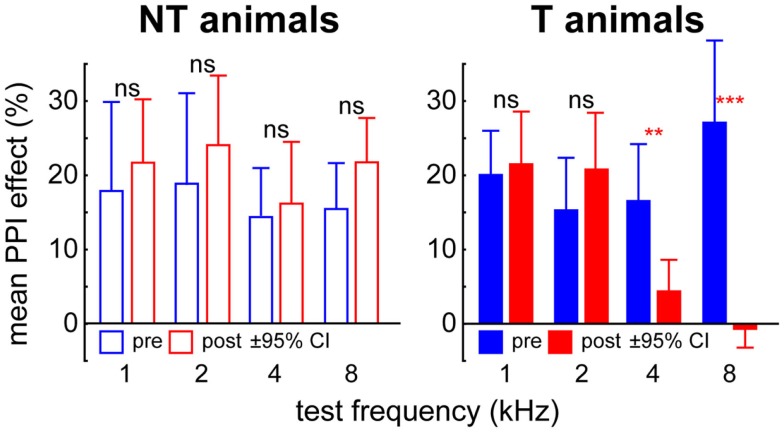
**Mean PPI effect (±95% confidence interval) in six exemplary animals classified as non-tinnitus (NT) and tinnitus (T) perceiving**. In the NT animals, PPI effect is always significant (single sample *t*-tests vs. 0) and no changes in PPI effect were found comparing pre (blue) and post trauma (red) data by *t*-tests. In T-animals this was only true for 1 and 2 kHz but not for 4 and 8 kHz test frequencies. Asterisks indicate the significance levels of the student’s *t*-tests pre vs. post trauma, **p < 0.01, ***p < 0.001.

**Figure 2:**
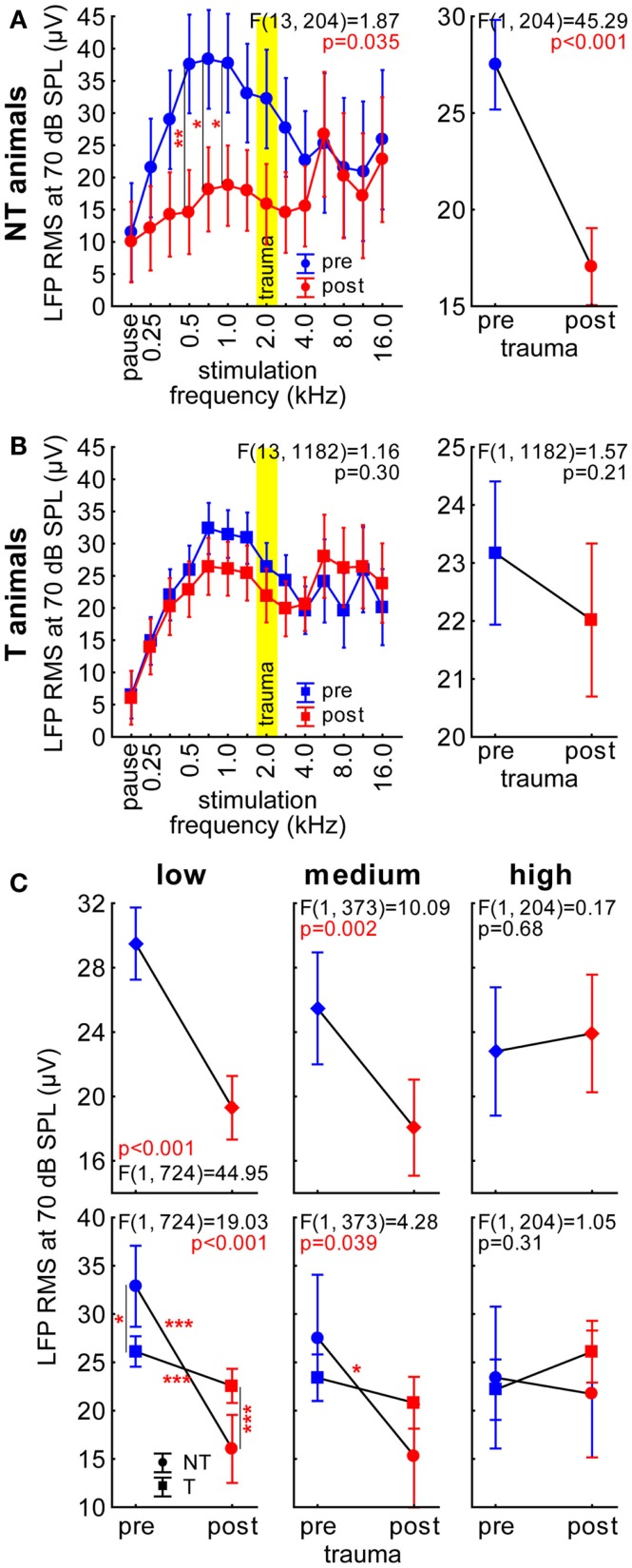
**Root mean square (RMS) values of the LFP recordings in AC with 70 dB SPL stimulation intensity**. **(A)** Results of the 2-factorial ANOVA of mean LFP RMS (±95% confidence interval) in NT animals; the left panel shows the interaction of the predictors stimulation frequency and time of measurement (pre-trauma: blue, post trauma: red), the trauma frequency (2 kHz) is indicated by a yellow bar. Asterisks indicate the significance levels of the Tukey *post hoc* tests, *p < 0.05, **p < 0.01. The right panel gives the comparison of time of recording averaged over all frequencies as part of the 2-factorial ANOVA. **(B)** Results of the 2-factorial ANOVA of LFP RMS in T-animals, symbols as in A. Note that we did not find any significant change over time and no interaction of the two predictors in these animals. **(C)** Comparison of LFP RMS in both animal groups over time by 2-factorial ANOVAs separated by stimulation frequency ranges (low: below 2 kHz; medium: 2–5.6 kHz; and high: above 5.6 kHz). Given in the upper panels are the temporal comparisons of the data averaged across both groups (diamonds) and in the lower panels its interaction in NT (circles) and T-animals (squares) before and after trauma; asterisks indicate the significance levels of the Tukey *post hoc* tests, *p < 0.05, **p < 0.01, ***p < 0.001.

For tinnitus testing we used two modified ASR paradigms in all animals before and after trauma. These consisted of either a 90 dB SPL pure tone startle stimulus of 1, 2, 4, or 8 kHz within a 50 dB SPL continuous white noise, or a 90 dB SPL click startle pulse within a 50 dB SPL band pass filtered noise with a center frequency of 1, 2, 4, or 8 kHz and a bandwidth of one octave [cf. Ref. (17)]. In both cases, a silent 15 ms gap within the noise (white or band pass) 100 ms before the startle stimulus served as pre-pulse. The rationale of these paradigms is that an animal that perceives a tinnitus would be impaired in gap detection because it would hear its own tinnitus within the silent gap [cf. Ref. (21)]. Consequently, when using a gap as pre-pulse, animals with tinnitus should produce smaller PPI of ASR than animals without tinnitus (cf. Figure 1). In humans, it is known that the majority of tinnitus percepts (around 70%) exhibit a pure tone-like character while the remaining part consists of ringing tones, noises or even more complex sounds (22). We used the two different paradigms to determine possible tinnitus percepts in the animals because the exact nature of these percepts is unknown. With the pure tone stimulus over a white noise and the click stimulus over band pass filtered noise we aimed to cover as many different percepts as possible. If a tinnitus is detected, the different tone frequencies and noise spectra used should give a rough estimate of the spectral content of the tinnitus percept. Each frequency and gap-condition was repeated 15 times and each test was performed before and after the acoustic trauma. Again the data obtained were checked by eye by the same two experimenters as above via a custom-made Matlab program. Trials in which the animals moved within 100 ms before the startle stimulus were discarded; in the valid trials only peak-to-peak amplitudes of responses within the first 50 ms after startle stimulus onset were used for further analysis (17). This approach allowed the determination of a possible frequency-specific tinnitus-related behavior at one octave precision. We tested the gap-effect on the response amplitude separately in each individual animal before and after trauma for each tested frequency by *t*-tests (α = 0.05) and found a significant PPI effect in all pre-trauma data (i.e., a reduction of startle amplitude in the condition with the gap in the background noise) and in each individual animal (*p* < 0.05). After the trauma only some animals showed an undisturbed gap-effect at all frequencies tested while other animals showed no gap-effect at some but not all frequencies (cf. Figure 1), which gave a first hint of a possible tinnitus percept but was not yet used as the final classification of the animals in the tinnitus or NT group (cf. below). To avoid possible effects of the acoustic trauma on different stimulation frequencies all startle response data were normalized to minimize variance of the response amplitudes. Normalization was performed as described earlier (17, 23); briefly, we divided each amplitude by the corresponding median amplitude of the 90 dB SPL only condition (which reflects the full startle response of the animal for the loudest condition at each specific frequency, 0 dB SPL pre-stimulus intensity Figure S1 in Supplementary Material). Thus we attempted to control for differences in the startle amplitudes resulting from the hearing loss at the trauma frequency. This normalization also guarantees that the reduced ASR response after acoustic trauma is not due to hearing loss but likely due to a tinnitus percept (23). Finally, the PPI of ASR in the healthy animal (“pre-trauma”) and after the trauma was calculated and the change in PPI relative to pre-trauma (in %) was tested against 0 (no change) for each frequency separately by *t*-tests (α = 0.025). Significant positive values for PPI change reflect impaired PPI and therefore indicate the development of a tinnitus percept. Only such animals were therefore classified as probably perceiving a tinnitus (T group) that showed at least one impaired frequency after the trauma independent of the affected frequency itself. As it turned out, in all cases where a tinnitus was detected according to one of the two gap-ASR paradigms used, the second gap-ASR paradigm was also positive for tinnitus. Only the frequencies affected could differ between gap-ASR paradigms. Animals without such a significant increase in PPI change were classified as NT perceiving animals (NT group). It became apparent that animals classified as T or NT based on these behavioral measures also differed in neurophysiological response measures (ABR and AC responses; cf. e.g., Figure 2), thereby strengthening the classification [cf. also Ref. (24)]. While the tests used here may only detect acute tinnitus, exemplary animals were re-tested in regular intervals for up to 16 weeks and did not show any changes in tinnitus frequencies. Based on these results we think that the classification of NT and T-animals is not only valid in acute but also in the chronic state of the disease.

Auditory brainstem responses data were obtained under anesthesia (mixture of ketamine, xylazine, NaCl, and atropine at a mixing ratio of 9:1:8:2, initial dose: 0.3 ml subcutaneous; Anesthesia was kept constant via a subcutaneous infusion with 0.2–0.3 ml/h.) via three subcutaneously placed thin silver wire electrodes, one for grounding at the back of the animals, one reference electrode at the forehead between the eyes and the ears and the measuring electrode infra-auricular overlying the bulla. Signals were recorded using a Plexon Multichannel Acquisition Processor (Plexon Inc., Dallas, TX, USA) after amplification by a JHM NeuroAmp 401 (J. Helbig Messtechnik, Mainaschaff, Germany; bandpass filter 400–2000 Hz, amplification 10,000) and stored with a custom-made Matlab program (at 10 kHz sampling rate). Auditory stimuli were generated by a custom-made Matlab program and presented free field to one ear at a time via a frequency transfer function corrected speaker (SinusLive neo 25S, pro hifi, Kaltenkirchen, Germany) at circa 0.5 cm distance from the animal’s pinna while the contralateral ear was tamped with an ear plug; both ears were measured directly after each other without any predefined order. Stimuli presented were clicks (0.1 ms duration) and pure tones (4 ms duration including 1 ms cosine-squared rise and fall times) ranging from 0.5 to 16.0 kHz in half-octave steps with 120 repetitions each. Stimulation was pseudo-randomized using a fixed list of all combinations of stimulus frequencies and sound pressure levels (0–90 dB SPL in 5 dB steps). To obtain ABR-based audiograms the mean ABR wave amplitudes were compared to the mean amplitude 200–100 ms before the stimulus (baseline). Thresholds were defined automatically by a custom-made Matlab program at the highest attenuation at which the evoked amplitude raised over two standard deviations of the baseline; data were discarded at frequencies where this procedure was not possible, e.g., at low signal to noise ratios. For additional analysis the root mean square (RMS) value of the ABR signal was calculated from 1 to 5 ms after stimulus onset and single waves of the ABR responses representing the different levels within the brainstem (25) were detected by a custom-made Matlab program at 70 dB SPL for each stimulation frequency. According to our classification the identified waves were: wave I/II (nervus cochlearis and nucleus cochlearis), wave III (superior olivary complex), wave IV (lateral lemniscus), and wave V (IC). All data were analyzed statistically with Statistica (StatSoft Inc., Tulsa, OK, USA)

### Electrophysiological recordings of LFP in primary AC

Of the 35 animals investigated behaviorally, 20 were used for electrophysiological recording in brainstem and AC. Among these, the complete recording protocol in AC could be accomplished in four T and two NT animals. In total 129 locations in AI were sampled to record LFP. The recordings were identical to those described in the earlier report (17) allowing us to additionally analyze single unit responses within AC. Describing briefly the approach of electrophysiological recordings in AC, two to five days after obtaining baseline ASR and ABR data, i.e., before the acoustic trauma, the skull of the anesthetized animals was trepanned to expose the left AC. A 2.5 cm aluminum head-post and a recording chamber were implanted. Recording began two to four days after surgery. Animals were again ketamine-xylazine anesthetized, placed on a 37°C warm heating pad and fixated via the aluminum head-post. Over two to three sessions every second day, LFP responses (0.7–300 Hz band pass filter) to pure tones in five to seven tracks with two to four recording depths each were recorded in AC using acutely inserted tungsten microelectrodes (1 MΩ impedance, 1-2 μm tip diameter, Plexon microelectrodes PLX-ME-W-3-PC-3-1.0-A-254). Auditory stimulation consisted of pure tones (200 ms including 1 ms cosine-squared rise and fall times) ranging from 0.25 to 16.0 kHz in quarter-octave or half-octave steps presented at 70 dB SPL with 500 ms interstimulus intervals. The recorded LFPs were analyzed with custom-made Matlab programs and statistically evaluated with Statistica. Three response measures were evaluated, first, the amplitude and timing of the first negative peak (N1) and the second positive peak (P2) waves of the recorded data (reflecting mainly the slower extralemniscal and intracortical input [for review see Ref. (26)], second, the RMS values of the LFP over the first 80 ms after stimulus onset and, third, the LFP frequency spectrum based on fast Fourier analysis (FFT).

### Statistical analysis and grouping of the data

We statistically analyzed behavioral and neuronal response amplitude data as well as FFT frequency spectra by parametric tests; response latencies and durations were assessed by non-parametric tests as one cannot assume normal distribution for these parameters. The grouping of the data was the same as in our earlier study with T and NT animals’ data separated by their behavior and stimulation frequency ranges defined by the changes in hearing threshold with *low frequencies* ranging from 250 Hz to 1414 Hz (frequencies below the effects of the acoustic trauma), *medium frequencies* ranging from 1682 Hz to 5656 Hz (frequencies affected by the acoustic trauma) and *high frequencies* above 5858 Hz to 16000 Hz (frequencies above the effects of the acoustic trauma).

In most parametric analyses we display the mean response amplitudes (±95% confidence intervals, CI) by the interaction of two predictors derived from a 2-factorial ANOVA with or without one or both effects of the single factors. Most analyses are separated for a third factor (e.g., stimulation frequency range). In the case of Fourier transformed data, we show heat map plots for all sound intensities over the different response frequencies separated for animal group and time of measurement. Statistically, we concentrated on the data at the moderate 70 dB stimulation intensity level by 2-factorial ANOVA. Tukey *post hoc* tests were used to further asses the differences in the data within one ANOVA. As mentioned above, response latency and duration were assessed by non-parametric Kruskal–Wallis ANOVAs (*post hoc* test: multiple comparison of mean ranks) or Mann–Whitney *U*-tests. The median values and interquartile ranges of the data of both animal groups are usually displayed in the same plots for easier comparison while analyses were performed separately.

## Results

### Behavioral results

We here present a short overview of the behavioral results and ABR hearing thresholds, for a complete analysis of the data the reader is referred to reference (17). The hearing threshold was assessed not only under anesthesia by ABR but also in the awake animal by PPI of ASR. To make sure that the startle reflex we utilized for our measurement was not prone to habituation we calculated 1-factorial ANOVAs of response amplitudes as a function of the trial number. This was exemplarily done in the six animals used for cortical recordings. We analyzed the data separately for each tested frequency and trauma status (pre or post trauma) in the 0 dB SPL pre-stimulus condition. With one exception at 4 kHz in the post trauma condition (*p* = 0.049) we did not see any significant effects of the trial number on the response amplitudes. Additionally, in the same animals we did see the typical significant mean response amplitude decrease with increasing pre-stimulus intensity (Figure S1 in Supplementary Material; *p* < 0.001 in all 12 1-factorial ANOVAs). This was also true for all 35 animals on the individual and group level.

From the 35 animals used in this study 26 (74%) showed an impairment of PPI of ASR in the gap-noise paradigms for at least one frequency 7 days after the trauma compared to the healthy state. Therefore these animals presumably perceived a subjective tinnitus and were grouped into the tinnitus (T) group. The remaining nine animals did not show such impairment in PPI and therefore presumably did not perceive a tinnitus until seven days after the trauma. These animals were grouped into the NT group. Exemplarily the mean PPI effect (i.e., the reduction of the response amplitude by the perception of the preceding gap in the background noise) of the above mentioned six animals is depicted in Figure 1. The two NT animals did always (pre and post trauma) show a significant reduction of ASR (single sample *t*-tests vs. 100%, always *p* < 0.05) and no significant change in PPI effect between pre and post trauma (*t*-tests, always *p* > 0.05). The four T-animals did also show a significant reduction of ASR before the trauma but not for all frequencies after the trauma (4 and 8 kHz, single sample *t*-tests vs. 100%, *p* > 0.05) and the PPI effects also showed significant changes after the trauma for these two frequencies. Note that these differences are only taken as hints for a possible tinnitus percept, the final classification was made on the basis of the normalized individual PPI change relative to pre-trauma condition (cf. Materials and Methods; α = 0.025) but was consistent with the *t*-test group results in these six cases. All 35 animals showed a significant NIHL with similar ABR- and behavioral audiograms [cf. Figure 5 in Ref. (17)]. We also analyzed the NIHL of both animal groups (T and NT) dependent on the stimulation frequency by 2-factorial ANOVAs and found neither in the ABR nor in the behaviorally determined thresholds any frequency-specific differences between the animal groups.

### Electrophysiological recording of LFP in AC

Local field potential recordings were obtained from a total of 69 locations before (NT: 13; T: 56) and 60 locations after the acoustic trauma (NT: 12; T: 48) with single tungsten electrodes. The RMS values of the first 80 ms post stimulus onset at 70 dB SPL were calculated and analyzed, first, by 2-factorial ANOVAs with the factors *stimulation frequency* and *time* (pre or post trauma) for NT and T-animals separately and, second, by 2-factorial ANOVAs with the factors *time* and *group* (NT or T) for frequency ranges separately (for an example refer to Figure S2 in Supplementary Material). We used this analysis as a first approach to investigate the full response amplitude of the LFP signal rather than one specific wave or frequency band [for review see Ref. (27)]. Figure 2A depicts this analysis for the NT animals with the interaction of those two factors on the left panel and the factor *time* on the right panel. Both show highly significant differences between pre and post trauma measurements (statistics see Figure 2A), which is also true for the factor *stimulation frequency* [*F* (13, 204) = 3.50, *p* < 0.001]. Tukey *post hoc* tests of the interaction of the two factors revealed a significant decrease of LFP amplitude at 500, 707, and 1000 Hz after the trauma compared to the healthy state. In T-animals, the amplitude of the LFPs only showed a significant effect of *stimulation frequency* [*F* (13, 1182) = 17.44, *p* < 0.001] but not of *time* and no significant interaction (Figure 2B). These results are highly similar to the single- and multi-unit activity reported earlier for the same T and NT animals (17). Analogously to the methods in that report we here merged the data of frequencies below trauma frequency (low), around trauma frequency (medium), and above trauma frequency (high) and compared both animal groups before and after trauma with each other (Figure 2C). Comparing factor *group* (not shown) we found no difference in any of the frequency ranges [low: *F* (1, 724) = 0.006, *p* = 0.94; medium: *F* (1, 373) = 0.086, *p* = 0.77; high: *F* (1, 204) = 0.33, *p* = 0.57]. For the factor *time*, we found significant differences for low and medium frequencies with the post trauma LFP amplitudes being lower than in the healthy state. For the high frequency range, we did not find significant differences (Figure 2C, upper panels). Results on the interaction of the two factors were similar (Figure 2C, lower panels), Tukey *post hoc* tests showed additionally significant differences already before the acoustic trauma between NT and T-animals for low frequencies and a significantly larger change after the trauma in NT animals than in T-animals for low and medium frequencies. There was no interaction or significant change over time for high frequencies. Again, these findings show a close correlation between LFP amplitude measurement and neuronal spiking activity published in our earlier work (17).

**Figure 3:**
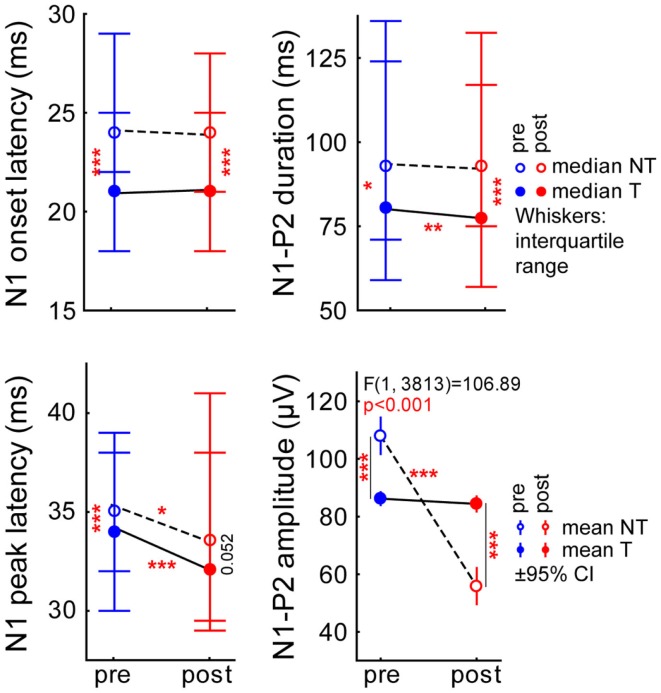
**Different parameters of N1 and P2 wave of the LFP response averaged across the sound intensities ranging from 50 to 90 dB SPL**. Averaged data across all frequencies: Median (±interquartile range) N1 onset latency (top left), N1 peak latency (bottom left), N1-to-P2 duration (top right) for NT (open symbols, broken line), and T-animals (filled symbols, solid line) before (blue) and after (red) the acoustic trauma; asterisks indicate the significance levels of the Mann–Whitney *U*-tests, *p < 0.05, **p < 0.01, ***p < 0.001. The bottom right panel gives the mean peak-to-peak amplitude (±95% confidence interval) of N1-to-P2 in a 2-factorial ANOVA interaction plot (time point × group) with the asterisks indicating the significance levels of the Tukey *post hoc* tests, *p < 0.05, **p < 0.01, ***p < 0.001.

A more fine-grained analysis of the LFP data, especially of the first negative peak (N1) and the second positive peak (P2) offers insight into preferential contributions of early and later cortical input (26). Figure 3 shows the results for the data averaged across all stimulation frequencies and intensities. The median onset latency of N1 (top left panel) did not show any significant changes after the trauma in both animal groups (pre vs. post trauma, Mann–Whitney *U*-tests: NT animals: *p* = 0.06, T-animals: *p* = 0.17), but we did find a significant difference in the overall level between animal groups with the T-animals showing a small but significant difference in onset latency with lower values compared to NT animals (Mann–Whitney *U*-tests: pre-trauma: *p* < 0.001, post trauma: *p* < 0.001). The peak latency of the N1 (Figure 3, bottom left panel) did show a significant small decrease in both animals groups after the trauma (Mann–Whitney *U*-tests: NT animals: *p* = 0.02, T-animals: *p* < 0.001) but only pre-trauma values of NT and T-animals showed a slight but significant difference (Mann–Whitney *U*-test, *p* < 0.001) while post trauma median values only showed a tendency for being different in the two animal groups (Mann–Whitney *U*-test, *p* = 0.052). The comparison of pre and post trauma median duration of the N1-P2 complex (Figure 3, top right panel) was different in T-animals only (Mann–Whitney *U*-test, *p* = 0.002) but not in NT animals (Mann–Whitney *U*-test, *p* = 0.17) while the difference between both groups was again small but significant at both time points with shorter durations in the T-animals (Mann–Whitney *U*-tests, pre: *p* = 0.02, post: *p* < 0.001). Finally, N1-to-P2 amplitude mean values were analyzed parametrically by a 2-factorial ANOVA. In general, noise trauma lowered the N1-to-P2-amplitude as the significant factor *time* revealed [mean ± standard deviation: pre: 97.1 ± 51.2 μV, post: 70.1 ± 50.5 μV; *F* (1, 3813) = 123.01, *p* < 0.001] while factor *group* was not significant [NT: 81.6 ± 45.1 μV, T: 85.4 ± 51.3 μV; *F* (1, 3813) = 1.94, *p* = 0.16]. The highly significant interaction of both factors (Figure 3, bottom right panel) [*F* (1, 3813) = 106.89, *p* < 0.001] indicates a strong dependency of the peak-to-peak LFP amplitude on *group* and *time point*. Tukey *post hoc* tests showed no change of amplitude in the T-animals after the trauma while NT animals showed a strong amplitude decrease (*p* < 0.001) pre vs. post trauma: while N1-to-P2 amplitudes were significantly higher in NT compared to T-animals before the trauma they dropped to lower values after the trauma (*p* < 0.001 for both). Taken together, we found significant differences between the two groups in all LFP parameters already before the trauma and in most parameters a parallel shift of parameter values after the trauma except for the N1-to-P2 amplitude, where only NT animals showed a significant decrease that inverted the N1-to-P2 amplitude relation between the two groups.

**Figure 4:**
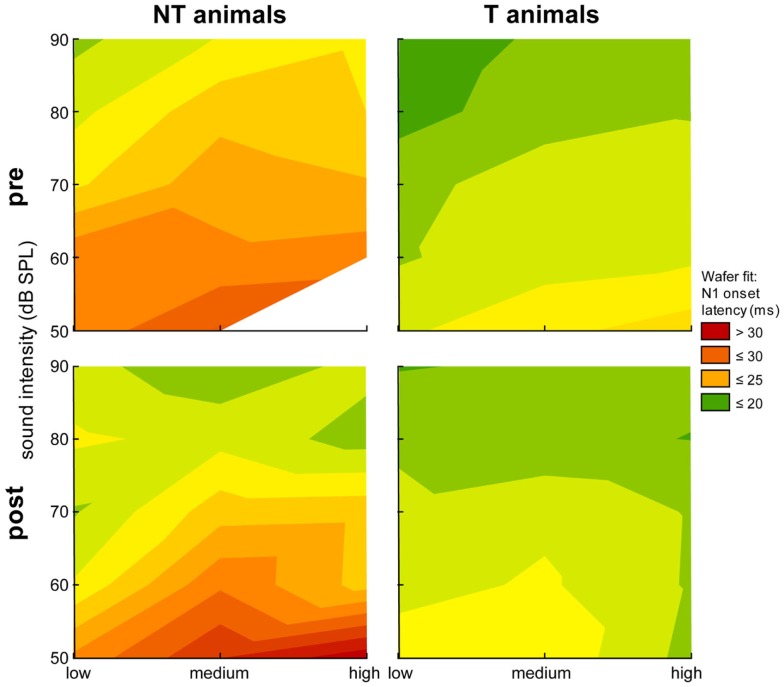
**Heat map plot of N1 onset latency for stimulation intensities ranging from 50 to 90 dB SPL against the three stimulation frequency ranges**. Data are separated for group (NT and T-animals) and time point (pre and post trauma). The median onset latencies are fitted by a Wafer fit, i.e., only the raw data are plotted in a direct format without any interpolation. The surface is constructed by creating triangles between the individual points, red colors indicate long latencies (above 30 ms) and green colors indicate short latencies (20 ms).

Figure 4 shows the relation of the N1 onset latency and stimulation frequency depending on different sound intensities plotted in a heat map. We found significant dependencies (Kruskal–Wallis ANOVAs) of the onset latency on group, time point, stimulation frequency, and sound intensity with longer latencies for lower stimulation intensities and higher stimulation frequencies in both animal groups after the trauma. For example, we did not find significant differences in the median onset latency for low medium and high stimulation frequency ranges for stimulation intensities of 50 dB SPL in NT animals neither before [*H* (1,9) = 2.46, *p* = 0.12] or after the trauma [*H* (2,28) = 2.05, *p* = 0.39]. This was also true for the single Mann–Whitney *U*-tests performed between pre and post trauma onset latencies for each stimulation frequency range. On the other hand, Kruskal–Wallis ANOVAs became significant for these animals for stimulation intensities of 90 dB SPL before trauma [*H* (2,66) = 9.63, *p* = 0.008] but did not show a significant difference between onset latencies of the three different stimulation frequency ranges after the trauma [*H* (2,48) = 3.42, *p* = 0.18]. Generally, in the NT animals we found significant dependencies of the onset latency on stimulation frequency only at the two most intense stimulation levels (80 and 90 dB SPL) before the trauma, after the trauma these dependencies did show up in the intermediate levels of 60 and 70 dB SPL. Contrary to this, in T-animals we found significant dependencies of the onset latencies over all loudness levels already before the trauma [e.g., 50 dB: *H* (2,373) = 21.30, *p* < 0.001; 90 dB: *H* (2,307) = 44.73, *p* < 0.001] and for the three lowest intensities (50–70 dB SPL) after the trauma [e.g., 50 dB: *H* (2,259) = 16.44, *p* < 0.001] but similar to the NT animals no significant dependency of onset latency in the loudest stimulation intensities [e.g., 90 dB: *H* (2,262) = 0.27, *p* = 0.87]. Similar results were found for the dependency of the N1 latencies on the stimulation frequency range. In general, the tendency to longer LFP latencies in NT animals that was seen in Figure 3 is again evident in the more detailed analysis in Figure 4.

**Figure 5:**
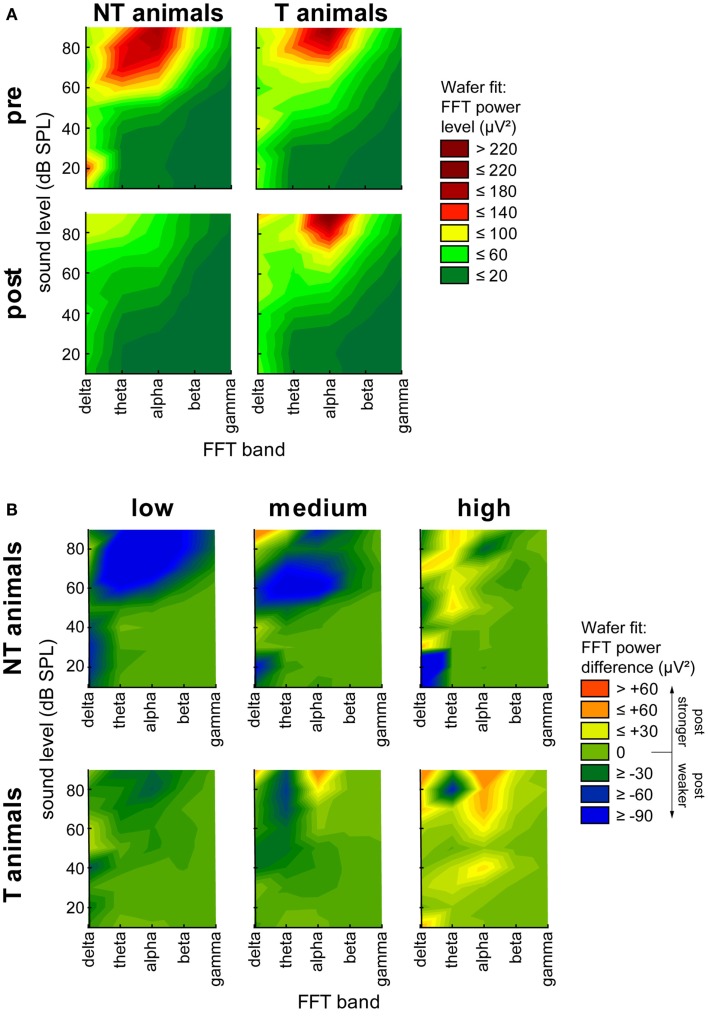
**Heat map plots of FFT power spectrum**. **(A)** Averaged spectral power (μV^2^) over all stimulation frequencies for all sound intensities plotted over all FFT power bands (delta, theta, alpha, beta, and gamma) separated for animal group and time point of measurement. Mean data are fitted by wafer fit with red colors indicate high spectral power (>220 μV^2^) and green colors indicate low spectral power (<20 μV^2^). **(B)** Difference between pre and post trauma spectral power data separated for low, medium, and high stimulation frequencies. Data are fitted by Wafer fit, red colors indicate an increase of power after the trauma larger than 60 μV^2^, green colors indicate no change, and blue colors indicate a loss of spectral power by more than 90 μV^2^ after the trauma.

**Figure 6:**
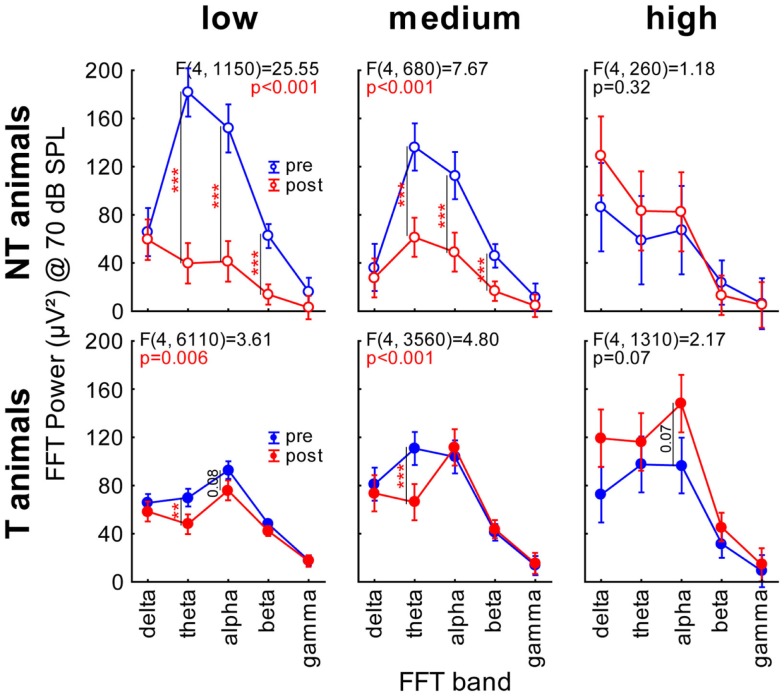
**Two-factorial ANOVAs interaction plots of spectral power at 70 dB SPL**. Given are the six interactions (FFT power band × time point) of the mean spectral power for both animal groups and the three different stimulation frequency ranges. Blue symbols and lines indicate the mean spectral power (±95% confidence interval) before, red after the acoustic trauma; asterisks indicate the significance levels of the Tukey *post hoc* tests, *p < 0.05, **p < 0.01, ***p < 0.001.

Finally, we subjected our LFP data to a FFT analysis. We grouped the data into the classical frequency bands, namely delta (0–4 Hz), theta (4–8 Hz), alpha (8–12 Hz), beta (12–28 Hz), and gamma (28–40 Hz), and plotted the FFT power spectra as a function of *time* (pre or post trauma), *group* (T or NT), and *sound intensity*. Figure 5A shows heat map plots of this analysis. Whereas the general pattern of LFP heat maps was comparable between all group and time combinations, NT animals showed overall higher levels of FFT power compared to T-animals before the trauma, but lower levels than T-animals after the trauma. A more detailed analysis of the change of power after the trauma separately for the three different ranges of stimulation frequencies (Figure 5B) revealed a strong decrease in spectral power in NT animals at low and medium frequency band stimulation above 60 dB SPL over a wide range of FFT frequency bands, but only small changes at high stimulation frequencies. In contrast, T-animals showed almost no changes in FFT power levels at low frequency band stimulation but an increase in alpha power for stimuli above 70 dB SPL at medium and above 30 dB SPL at high stimulation frequencies. We chose to investigate this change in power at 70 dB SPL stimulation intensity in more detail and performed 2-factorial ANOVAs for NT and T-animals in the three different stimulation frequency ranges. The results of the interactions of the factors *time point* (pre vs. post trauma) and *FFT frequency band* are shown in Figure 6 (for between group analyses for the single FFT frequency bands please refer to Figure S3 in Supplementary Material). The variance analysis and Tukey *post hoc* tests of the spectral power of the LPF data showed a strong decrease in the theta, alpha, and beta bands at low and medium stimulation frequencies in NT animals after the trauma, but no significant change at high stimulation frequencies. In T-animals, trauma induced changes in FFT power were in general smaller than in NT animals: there, we only found a small but significant decrease of power in the theta and alpha band after the trauma at low stimulation frequencies, a decrease in theta band power at medium stimulation frequencies and a tendency for an alpha band power increase at high stimulation frequencies [mean post trauma power (88.59 μV^2^)] is significantly increased compared to mean pre-trauma power [61.38 μV^2^, *F* (1, 1310) = 17.99, *p* < 0.001]. In summary, these FFT analyses showed results comparable to the RMS analyses of the LFP signal, namely a more general decrease in activity after the acoustic trauma in NT animals compared to subtle changes in T-animals that were mostly restricted to higher stimulation frequencies, corresponding to the behaviorally determined frequency range of the tinnitus percept.

### Electrophysiological recording of ABR

**Figure 7:**
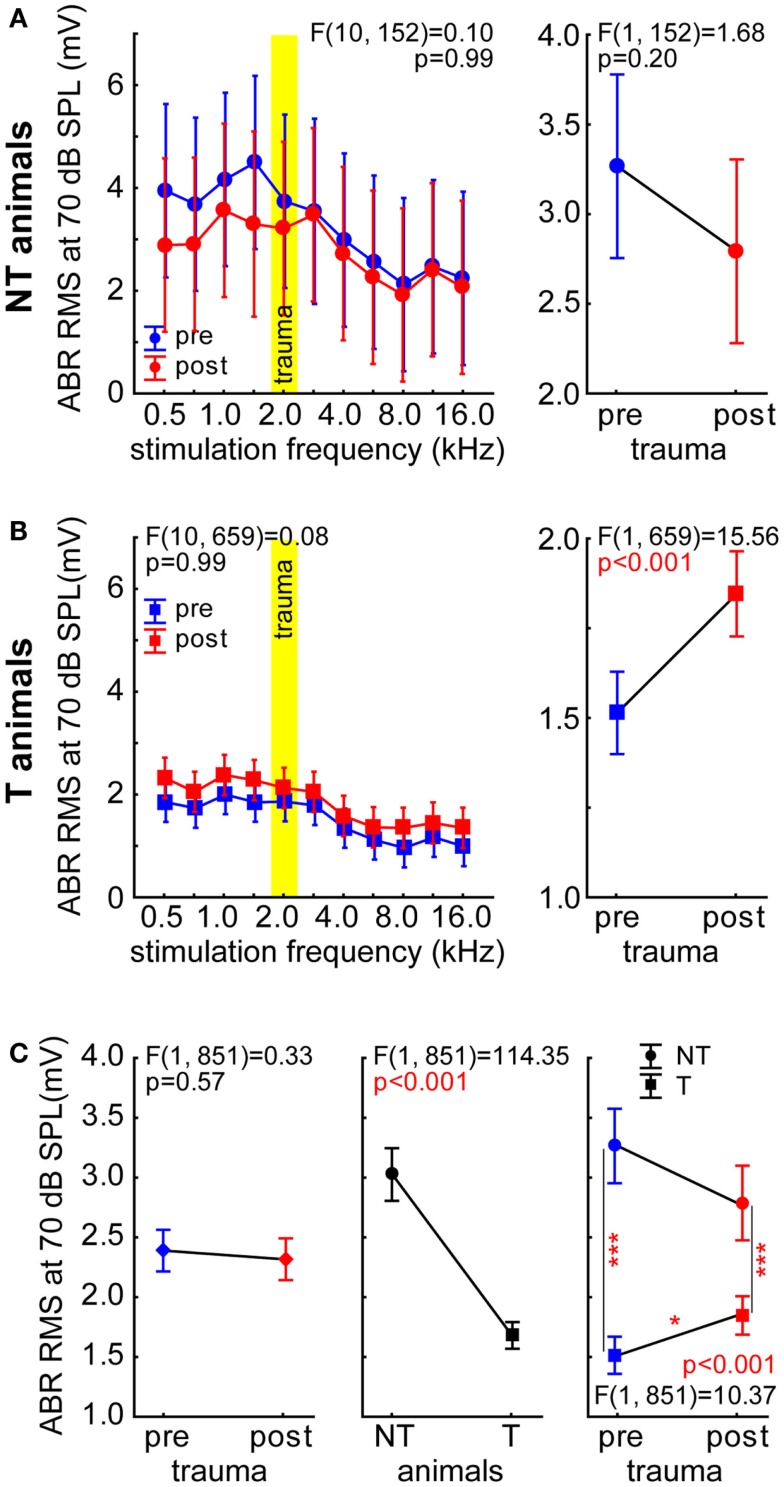
**Root mean square values of the ABR measurements at 70 dB SPL stimulation intensity**. **(A)** Results of the 2-factorial ANOVA of mean ABR RMS (±95% confidence interval) in NT animals, symbols as in Figure 1. **(B)** Results of the 2-factorial ANOVA of mean ABR RMS (±95% confidence interval) in T-animals. Note that only in this group a significant change (increase) of ABR RMS over time could be found. **(C)** Comparison of ABR RMS in both animal groups over time by 2-factorial ANOVAs, asterisks indicate the significance levels of the Tukey *post hoc* tests, *p < 0.05, ***p < 0.001.

We recorded ABR signals with sound intensities ranging from 0 to 90 dB SPL and calculated the RMS values of the amplitudes within the first 5 ms, i.e., the activity within the auditory pathway from the auditory nerve fibers to the IC. In this report, we focused on ABR to tones presented with 70 dB SPL as this was the sound intensity used for cortical recordings. In Figure 7, the 2-factorial ANOVAs of the RMS values of the ABR measurements with the factors *stimulation frequency* and *time of recording* are shown separately for NT animals (Figure 7A) and for T-animals (Figure 7B). In NT animals, we did not see any significant changes in ABR RMS values when comparing pre vs. post trauma recordings [*F* (1, 152) = 1.68, *p* = 0.20] nor did we find any significant frequency dependency [*F* (10, 152) = 1.26, *p* = 0.26]. We also did not find any interaction of the two factors [*F* (10, 152) = 0.10, *p* = 0.99]. In the T-animals on the other hand, we did find a significant increase of RMS values after the acoustic trauma [*F* (1, 659) = 15.56, *p* < 0.001] and a dependency of ABR amplitude on stimulation frequency [*F* (10, 659) = 8.67, *p* < 0.001]. This shift in amplitude after the trauma was identical for all frequencies (i.e., parallel shift of function), as interaction did not become significant [*F* (10, 659) = 0.08, *p* = 0.99]. For comparison of both animals groups we averaged all ABR RMS values at 70 dB SPL and performed a 2-factorial ANOVA with the factors *time of measurement* and *group*. This analysis is shown in Figure 7C, no change over time in ABR RMS could be found [left panel; *F* (1, 851) = 0.33, *p* = 0.57] but a strong difference between the groups with NT animals having significantly larger ABR amplitudes than T-animals [middle panel; *F* (1, 851) = 114.35, *p* < 0.001]. The interaction of both predictors was also significant [right panel; *F* (1, 851) = 10.37, *p* < 0.001], indicating different changes in NT and T-animals over time. This became even more clear with Tukey *post hoc* tests showing significant differences between NT and T-animals at both times of measurement (*p* < 0.001 each) but only a significant change (increase) of ABR RMS values over time in T-animals (*p* = 0.018).

**Figure 8:**
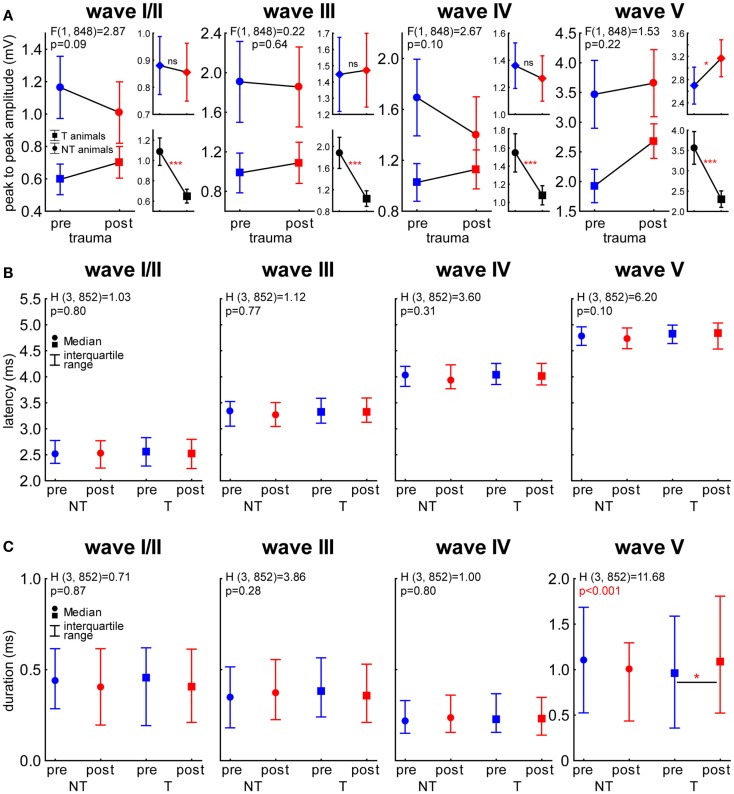
**Single wave analysis of ABR in NT and T-animals**. **(A)** 2-factorial ANOVA of peak-to-peak amplitudes (±95% confidence interval) over time and animal groups for the waves I/II to V. Larger panels show the interaction of both predictors and smaller panels the temporal (upper panels) and group effects (lower panels). **(B)** Kruskal–Wallis ANOVAs of the median latency (± interquartile range) of each wave in the different animal groups and time points. Note that we did not see any differences in latency. **(C)** Kruskal–Wallis ANOVAs of the median duration (± interquartile range) of each wave in the different animal groups and time points. Note that only T-animals showed a significant prolongation of wave V (inferior colliculus) after the trauma.

To investigate if the differences in general ABR amplitudes, as assessed by RMS values, are dependent on one or more auditory brainstem nuclei we investigated each ABR wave separately [with wave I and II merged as usually performed in rodent ABR wave analysis (25)] with respect to its peak-to-peak amplitude as well as its latency and duration. These data were compared with a 2-factorial ANOVA (amplitude) and Kruskal–Wallis ANOVAs (latency and duration). In Figure 8A, the results of the amplitude comparisons for the different ABR waves are shown. Generally, we found NT animals always showing larger amplitudes than T-animals (*p* < 0.001 for NT vs. T in all waves) but no changes over time in wave I to IV could be found. In wave V, however we did see a significant increase of the amplitude after the trauma [*F* (1, 848) = 4.23, *p* = 0.04] but neither there nor in the other waves we found a significant interaction of both predictors (statistics see Figure 8A). In Figure 8B, the latency of the waves I/II to V are shown. Between the groups and over time we did not see any significant latency changes within each wave (Kruskal–Wallis statistics see panels). This was also true for the wave duration (Figure 8C) of wave I/II to IV, but in wave V a significant duration difference was indicated by the analysis: Multiple comparison of mean ranks *post hoc* test showed that only in T-animals a significant increase of wave V duration after the trauma could be found (*p* = 0.016).

**Figure 9:**
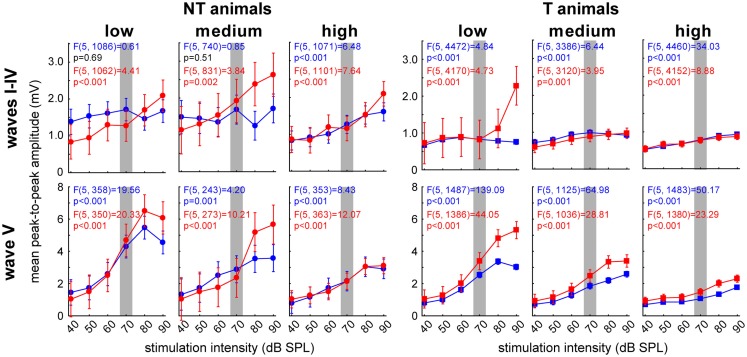
**Detailed analysis of peak-to-peak amplitude-intensity functions of ABR wave V in comparison to averaged waves I–IV**. Given are the 12 1-factorial ANOVAs of wave amplitudes (means ± 95% confidence intervals) as a function of stimulation intensity (40–90 dB SPL) for both animal groups (NT and T) and wave class (wave I–IV and V, respectively) separated for stimulation frequency range. The gray bar indicates the stimulation intensity at which the analysis of cortical LFPs was based.

These results seem to point to the IC (ABR wave V) as one of the central subcortical candidate structures for the temporal and group effects found in NT and T-animals. Because of this we chose to investigate the amplitudes of this wave in more detail and averaged the other waves for comparison. Analog to the analyses of the LFP signals we separated the responses into low, medium, and high frequency domains and analyzed pre and post trauma amplitude-intensity-functions by 1-factorial ANOVAs (Figure 9). Interestingly, we again found differences between NT and T-animals in the response characteristics of the different waves especially before the trauma, i.e., in the healthy animals (blue). In healthy NT animals, the major effect of stimulation intensity was seen in the IC for most frequencies except the highest (frequencies above 5.6 kHz) as demonstrated by the significant wave V increase of amplitudes there that was not seen in wave I-IV (Figure 9, left panels: low and medium frequencies; 1-factorial ANOVAs show dependency/lack of dependency of amplitude on sound intensity). This was not the case in healthy T-animals as here intensity effects were seen in all frequency domains for wave I–IV and wave V, while still the strongest responses were found for wave V (Figure 9, right panels: blue curves). Notice the overall difference of absolute amplitudes in healthy NT and T-animals, with the NT animals showing significantly higher mean amplitudes at all frequency domains and ABR waves (*t*-tests NT vs. T, always *p* < 0.05).

After the acoustic trauma the ABR peak-to-peak response wave amplitudes showed different changes dependent on the animal group and stimulation frequency. NT animals’ wave I–IV for low and medium frequencies (Figure 9, left upper panels: red curves) showed intensity-dependent effects with decreased responses at lower intensities (low frequencies: *t*-tests pre vs. post for 40 and 50 dB, *p* < 0.05) and increased responses especially at higher stimulus intensities above 70 dB (medium frequencies: *t*-tests pre vs. post for 80 and 90 dB, *p* < 0.05). This increase at higher stimulus intensities was also seen at responses to low (*t*-test pre vs. post for 90 dB, *p* = 0.05) and medium frequencies (*t*-test pre vs. post for 90 dB, *p* < 0.05) in wave V (Figure 9, left lower panels, red curves). Both changes could not be seen in the high frequency domain of NT animals.

T-animals’ responses also showed a strong amplitude increase in wave I–IV after the trauma for intense stimuli in the low frequency domain (*t*-tests pre vs. post for 80 and 90 dB, *p* < 0.05) but no decrease for low stimulus intensities and no changes in the medium and high frequency domains (Figure 9, right upper panels: red curves). In the post trauma wave V responses of the T-animals (Figure 9, right lower panels: red curves) we found a general increase of response amplitudes for all intensities and frequency domains (*t*-tests pre vs. post, always *p* < 0.05).

Taken together these results point to a general difference in stimulus intensity processing dynamics in the auditory brainstem in the two animal groups already in the healthy state: NT animals showed main intensity effects in the IC while significant T-animals’ effects were also seen at auditory brainstem levels more peripheral than the IC. After the trauma also NT animals showed intensity effects at these sub-IC levels and seemed to be able to stabilize responses especially in the IC up to around 70 dB SPL. T-animals did show almost no systematic changes in the waves I–IV and showed a strong increase of IC responses.

**Figure 10:**
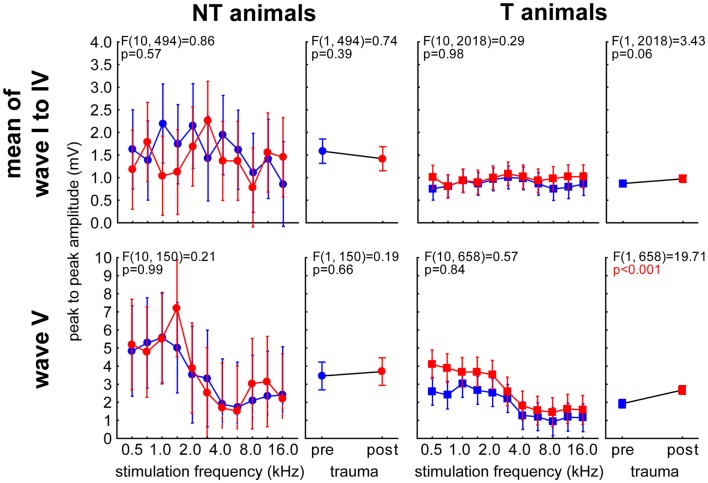
**Detailed analysis of peak-to-peak amplitude of ABR wave V in comparison with averaged waves I–IV at 70 dB SPL**. Given are the four 2-factorial ANOVAs (interaction and factor time) of wave amplitudes (means ± 95% confidence intervals) as a function of stimulation frequency and time of measurement for both animal groups and wave class. Frequency dependencies can only be found in wave V (lower panels) but not in earlier ABR waves (upper panels). Changes over time can only be seen in T-animals’ wave V.

Analog to the analyses described for the LFPs we performed 2-factorial ANOVAs with the factor *stimulation frequency* and *time of measurement* for the peak-to-peak amplitudes of both animal and wave groups (averaged wave I–IV vs. wave V). The main results of these analyses are depicted in Figure 10. NT animals (left panels) did not show a significant amplitude change over *time*. We also found no frequency dependency in the averaged wave group [wave I–IV; *F* (10, 494) = 0.75, *p* = 0.67] but a significant effect of *frequency* on the amplitudes of wave V [*F* (10, 150) = 3.09, *p* = 0.001]. In neither wave group did the interaction become significant. In T-animals (right panels), we found for the averaged waves I–IV a comparable result as in the NT animals, as the amplitudes did not change over *time* or *frequency* [*F* (10, 2018) = 0.53, *p* = 0.87] and also the interaction did not reach significance. However, the amplitudes of wave V in T-animals did show a significant increase after trauma, which therefore seems to explain the majority of the effect described in Figure 8B. Also the amplitudes of wave V of these T-animals were strongly dependent on *stimulation frequency* [*F* (10, 658) = 10.76, *p* < 0.001] but did not show any significant interaction of both factors, indicating a parallel shift of amplitude over frequency functions after the trauma (cf. also Figure 9). Taken together the frequency specificity of ABR amplitudes at 70 dB SPL seemed to rely mainly on wave V where we also see amplitude and response duration effects in animals that seemed to perceive a tinnitus percept (Figure 8). Likewise the absolute amplitude increase in T-animals predominately seemed to be based on wave V changes (cf. also Figure 9).

## Discussion

The data presented in this work are in line with our previous study describing the differences in neuronal plasticity between animals that develop a subjective tinnitus after noise trauma and those that do not (17). We reported that – at least in our animal model – there seems to be a predisposition for the development of NIHL-induced tinnitus that is evident in the overall neuronal output activity recorded in AC by means of extracellular single- and multi-unit recordings. Animals with comparably high overall evoked AC output activity were able to reduce this activity in response to noise trauma and subsequently did not develop a tinnitus percept. Those with comparably low overall AC output activity could not significantly suppress this activity in response to noise trauma and subsequently did develop a tinnitus percept. This finding is different from that reported by Rüttiger et al. (16) who also demonstrated a difference between T and NT animals. In their model, a difference before the noise trauma did not exist and the induced changes were rather depended on a different outcome of the trauma, i.e., the amount of inner hair cell ribbon loss and high frequency hearing impairment. A critical point in these studies is the detection of a possible tinnitus percept in the animals. We utilize the gap-in-noise-detection ASR method proposed by Turner and colleagues (20, 21, 28, 29) that is considered to be based on brainstem reflexes (30). A tinnitus percept is – as the term percept implies – a cortical phenomenon that is nevertheless known to be affected by processing along the whole auditory pathway (31, 32). Therefore influences on this percept may be measured using the gap-ASR method. As we performed these measures in the acute phase of a possible tinnitus development, we cannot rule out the possibility that some NT animals may develop a tinnitus percept at a later time point and NT animals hence may split into a late T and real NT group at a later point in time (33). Nevertheless, the tinnitus percept used for the behavioral grouping of T and NT animals was stable for up to 16 weeks, so that electrophysiological differences correlate with the time point of emergence of a long-lasting tinnitus percept.

With the present study we extended our earlier findings to synaptic input activity in AC as assessed by means of LFP recordings and also by presenting a more detailed analysis of ABR measurements. In general, our LFP data (Figure 2) qualitatively replicated the data on spiking responses in AC, i.e., the predisposition and plasticity described for AC output activity is also reflected in overall AC input activity. We further analyzed the N1-to-P2 complex of the LFP wave, which may reflect later stages of the input to the cortex, i.e., rather intracortical than thalamic input (26). The observation that T-animals in general showed lower LFP latencies and faster N1-to-P2 transition compared to NT animals (Figure 3) may indicate that responses in T-animals are dominated by bottom-up influences whereas in NT animals additional top-down influences are active that require more time and may be used to counteract the development of tinnitus after NIHL. A general bias of LFP amplitude on our measurement of LFP latency may be excluded as the latency difference between T and NT animals is still preserved after trauma, where the amplitude relation between T and NT animals is reversed (cf. Figure 3). Additionally, we find in the N1-to-P2 peak-to-peak amplitude very similar changes as seen with the general RMS approach with nearly no changes in T-animals and a strong decrease of amplitude in NT animals together with an obvious predisposition for post trauma tinnitus development, i.e., larger pre-trauma amplitudes in NT compared to T-animals. These new data therefore are also in line with our hypothesis that animals with high overall activity in AC are able to activate a global inhibitory mechanism to counteract the development of NIHL-induced tinnitus via top-down influences to the AC. This suppressive effect of such a top-down inhibitory mechanism may be used to equilibrate the overall activity in the auditory system that would otherwise be increased by a homeostatic response that tries to compensate for reduced cochlear input via an increased response gain (14, 15), leading to tinnitus in T-animals that lack or may not activate the top-down control mechanism. Further analyses of the power spectra of the LFP [e.g., Ref. (34, 35)] revealed a widespread power decrease in NT animals for intense stimuli in low and medium stimulation frequency ranges but only very specific changes in T-animals for roughly the same sound intensities, e.g., a trend for increasing power in the alpha band at high frequency stimulation. The results presented here are hence in part different to the results found in human resting MEG data, where spontaneous slow wave activity in the right temporal cortex was found to be increased in delta and decreased in alpha frequency bands in tinnitus patients (36) and also spontaneous gamma activity in the right temporal cortex was increased in these patients when compared with healthy controls (37). These differences possibly result from the different methods used. In the aforementioned studies, awake tinnitus patients’ resting activity was always compared to that of healthy controls, which presumably did not received any traumatic noise. We investigated anesthetized animals’ evoked activity in the left AC only and compared two groups of hearing loss affected individuals with and without tinnitus before and after NIHL. This approach allowed us to investigate the tinnitus development at a specific time point (the first week after NIHL), which is not possible in human research. Therefore, the differences between our and the human data may also be due to the time of recording relative to tinnitus induction (acute vs. chronic state).

Our data allow us to speculate about the origin of such a global inhibitory mechanism: the most striking electrophysiological attribute of the NT animals was the significantly reduced cortical activity at 70 dB SPL stimulation, but not of the overall ABR amplitudes in response to NIHL (Figure 2A vs. Figure 7A). Contrary, T-animals showed no significant change in cortical activity, but a significant increase in the overall amplitude of ABR (Figure 2B vs. Figure 7B). Furthermore this increase in ABR amplitude was frequency-unspecific (parallel shift of the ABR function in Figure 7B, left panel) and seems to be based primarily on changes in wave V representing the neuronal activity in IC (Figures 9 and 10). This finding therefore differs from those in humans, where an increase of wave III (trapezoid body – superior olivary complex) was reported in tinnitus patients (38) and no change in wave V could be found (15). Using a new method of current source density analysis (39), a preliminary report on changes in AC circuitry in our animal model (40) recently provided data pointing to a stronger recruitment of synaptic inputs in infragranular layers after NIHL. Furthermore, inputs in supragranular layers, mainly reflecting cortico-cortical processing, were altered. According to these observations it seems that changes in intracortical connections as well as cortico-efferent output projections to the thalamus and brainstem underlie the observed neuronal plasticity (cf. also Figure 9), rather than thalamo-cortical input connections to layer IV of the cortex. These considerations are in line with our observations in the LFP latencies (low and high frequency range changes, cf. Figures 3 and 4), LFP amplitudes (RMS and peak-to-peak, cf. Figures 2 and 3) as well as the drop within large parts of the power spectrum after the trauma in NT animals and specific increases in power in T-animals (cf. Figures 5 and 6). It therefore seems that the source of the global inhibition we propose either lies within intracortical circuit plasticity or outside the primary auditory pathway, possibly in prefrontal or limbic structures, but rather not in subcortical auditory centers. In awake animals, recruitment of additional inhibitory mechanisms might further contribute to circuit plasticity (41). In principle, as demonstrated earlier, the inhibitory circuitry in AC of the gerbil would allow long-range, global inhibitory interactions across the entire tonotopic gradient (42, 43). If activated, this global inhibition would reduce activity both in AC and IC, with the described differences in T and NT animals. A shortcome of this effect seems to be that it works well for lower and normal intensity stimuli in the brainstem but seems to fail for very intense stimuli (above 80 dB SPL) The tinnitus prevention mechanism proposed here therefore seems to be a “top-down” type of influence, rather than a plastic process conveyed via “bottom-up” circuits (cf. discussion on N1-to-P2 complex latencies above), as otherwise we would have expected to see changed inputs in thalamo-cortical input layer IV in AC [cf. Ref. (22)].

Finally, it is important to note that most current (bottom-up) models of tinnitus development [e.g., Ref. (44)] propose frequency-specific neuronal plasticity that depends on the exact spectral location and extent of the cochlear damage. Contrary, the tinnitus prevention mechanism proposed here is thought to act in a general manner by introducing inhibitory or suppressive effects across the whole spectral gradient via cortico-efferent plastic adaptations. Which one of these two mechanisms finally prevails may decide if a particular subject develops tinnitus after NIHL or not.

In conclusion, the current report extends the data presented in our earlier study on tinnitus predisposition and prevention (17) by providing electrophysiological recordings along the primary auditory pathway (ABR and LFP recordings in AC) in combination with a behavioral classification of early tinnitus development or resistance. Based on the results presented in both studies, we propose a model of tinnitus prevention after NIHL that acts via top-down global inhibition, reducing overall neuronal activity in AC, IC, and the lower auditory brainstem in a stimulus intensity-dependent but frequency-unspecific manner. This top-down mechanism may counteract NIHL-induced frequency-specific neuroplastic adaptations in bottom-up circuits to prevent the development of subjective tinnitus in a subgroup of individual, but not all animals. We identified the overall pre-trauma neuronal activity in the AC as a potential key determinant for such effective global top-down compensation.

## Conflict of Interest Statement

The authors declare that the research was conducted in the absence of any commercial or financial relationships that could be construed as a potential conflict of interest.

## Supplementary Material

The Supplementary Material for this article can be found online at http://www.frontiersin.org/Journal/10.3389/fneur.2015.00022/abstract
