# Supplementary material for: Noise Trauma Induced Neural Plasticity Throughout the Auditory System of Mongolian Gerbils: Differences between Tinnitus Developing and Non-Developing Animals
Source: Front Neurol. 2015 Feb 10;6:22. doi: 10.3389/fneur.2015.00022 (PMC4322711; doi:10.3389/fneur.2015.00022)
Supplement: Supplementary file 1 [file Data_Sheet_1.PDF]

Supplementary Figures

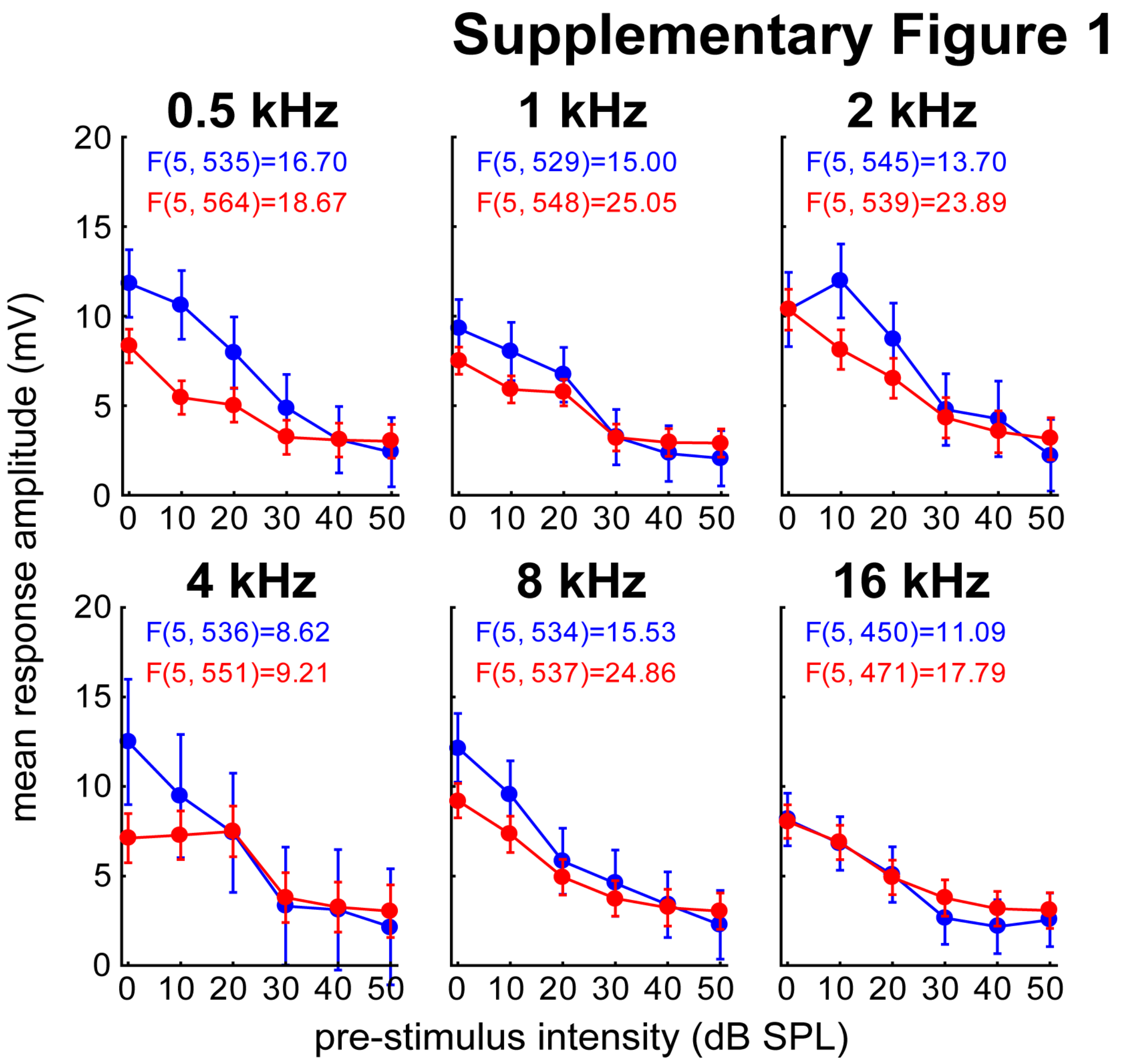

Supplementary Figure 1

Startle responses of six exemplary animals in the behavioral threshold measurements to all tested pre-stimulus intensities. In all twelve 1-factorial ANOVAs (means ± 95% confidence intervals; pre trauma = blue; post trauma = red) we find a significant (always p<0.001) reduction of response amplitude with increasing stimulus intensity.

Supplementary Figure 2

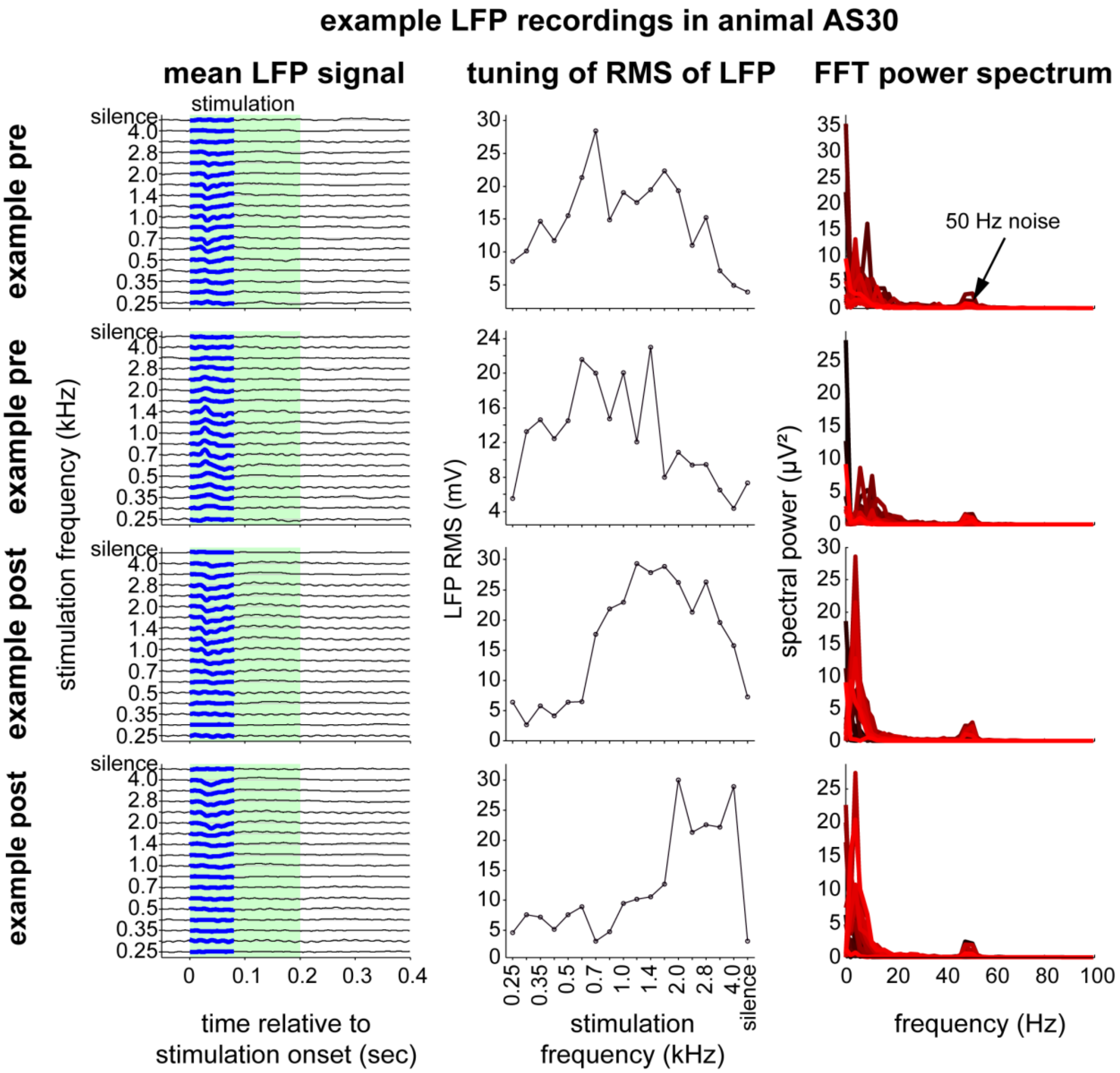

Supplementary Figure 2

Exemplary LFP waves of animal AS30, two recording sites pre (upper two rows) and two recording sites post trauma (lower two rows) are depicted. The left column shows the mean LFP signal of 15 repetitions each from 50 ms before to 400 ms after stimulus onset. The stimulus has a length of 200 ms (green area) and is a pure tone of different frequency. The blue section of the mean signal indicates the first 80 ms used for RMS calculation, which is shown in the central column. In the right column the spectral power of the complete signal for frequencies ranging from 0 to 100 Hz are given, every single line indicates the FFT power spectrum for one stimulation frequency, note that the majority of the power distributions lie below 20 Hz (except for the 50 Hz alternating current signal).

Supplementary Figure 3

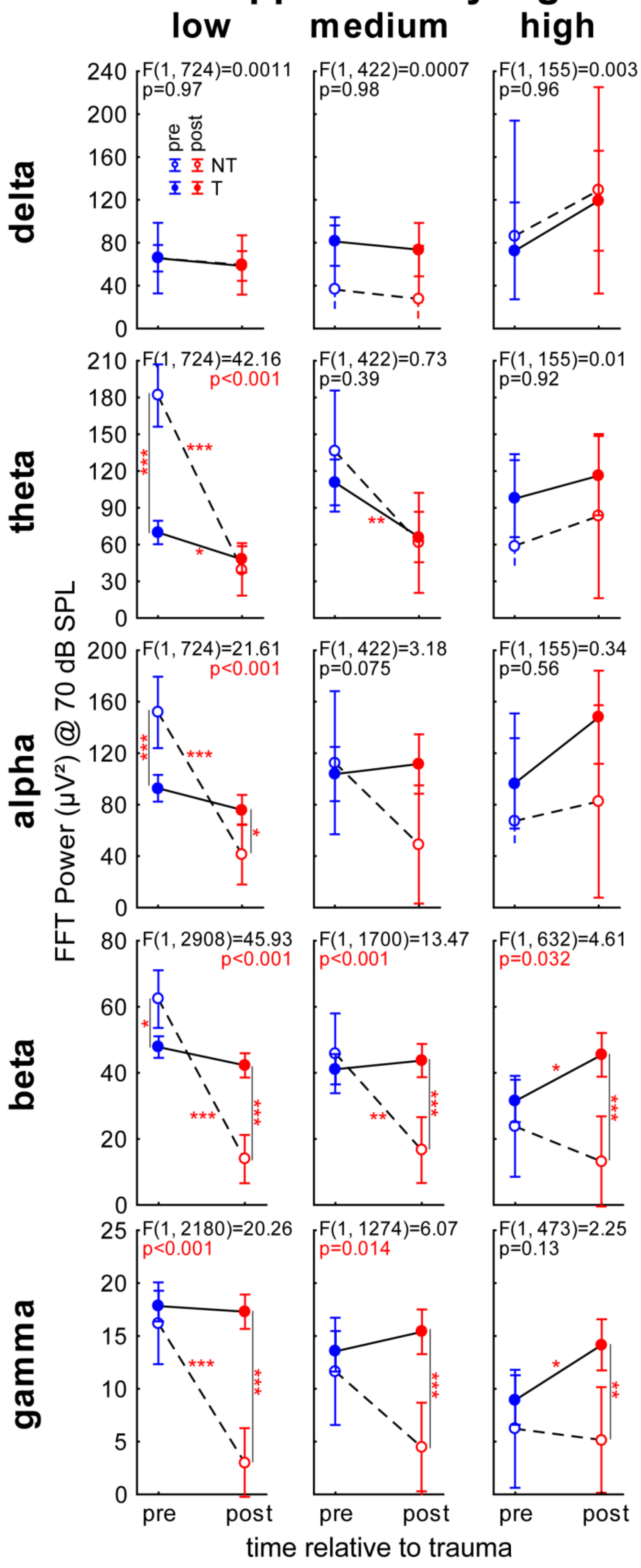

Supplementary Figure 3

2-factorial ANOVA interaction plots (time point x group) of FFT power at 70 dB SPL separated for stimulation frequency range and spectral frequency range. Data of the NT animals are depicted in open symbols and broken lines, T animals' data are given in filled symbols and solid lines, pre trauma data are blue and post trauma data red colored; asterisks indicate the significance levels of the Tukey post-hoc tests, * $p<0.05$, ** $p<0.01$, *** $p<0.001$. Please note the consistent power loss in the low frequency range over nearly all FFT frequency bands in NT animals. Contrary to this T animals show significant increases in beta and gamma frequencies in the high stimulation frequency range (corresponding to the tinnitus percept frequencies) after the trauma.
